# Synapsin I Controls Synaptic Maturation of Long-Range Projections in the Lateral Amygdala in a Targeted Selective Fashion

**DOI:** 10.3389/fncel.2019.00220

**Published:** 2019-05-21

**Authors:** Eleonora Lugarà, Antonio De Fusco, Gabriele Lignani, Fabio Benfenati, Yann Humeau

**Affiliations:** ^1^Department of Experimental Medicine, Section of Human Physiology, University of Genova, Genoa, Italy; ^2^Center for Synaptic Neuroscience and Technology, Istituto Italiano di Tecnologia, Genoa, Italy; ^3^IRCCS Ospedale Policlinico San Martino, Genoa, Italy; ^4^Team Synapse in Cognition, Institut Interdisciplinaire de Neuroscience, Centre National de la Recherche Scientifique CNRS UMR5297, Université de Bordeaux, Bordeaux, France

**Keywords:** synapsin, amygdala, synapse maturation, interneurons, cortical projections, thalamic projections

## Abstract

The amygdala, and more precisely its lateral nucleus, is thought to attribute emotional valence to external stimuli by generating long-term plasticity changes at long-range projections to principal cells. Aversive experience has also been shown to modify pre- and post-synaptic markers in the amygdala, suggesting their possible role in the structural organization of adult amygdala networks. Here, we focused on how the maturation of cortical and thalamic long-range projections occurs on principal neurons and interneurons in the lateral amygdala (LA). We performed dual electrophysiological recordings of identified cells in juvenile and adult GAD67-GFP mice after independent stimulation of cortical and thalamic afferent systems. The results demonstrate that synaptic strengthening occurs during development at synapses projecting to LA principal neurons, but not interneurons. As synaptic strengthening underlies fear conditioning which depends, in turn, on presence and increasing expression of synapsin I, we tested if synapsin I contributes to synaptic strengthening during development. Interestingly, the physiological synaptic strengthening of cortical and thalamic synapses projecting to LA principal neurons was virtually abolished in synapsin I knockout mice, but not differences were observed in the excitatory projections to interneurons. Immunohistochemistry analysis showed that the presence of synapsin I is restricted to excitatory contacts projecting to principal neurons in LA of adult mice. These results indicate that synapsin I is a key regulator of the maturation of synaptic connectivity in this brain region and that is expression is dependent on postsynaptic identity.

## Introduction

Emotions, fear and anxiety are mostly encoded in the cerebral formation of the amygdala both in humans and rodents (LeDoux, 2003). The lateral nucleus (LA) of amygdala, which is part of the basolateral amygdala (BLA) complex, is principally involved in processing sensory information coming from external circuits, especially from cortical and subcortical (thalamic) inputs (Bosch and Ehrlich, 2015). Two major neuronal populations implicated in fear learning and memory are present in the LA: principal neurons (PNs) representing the 80–90% of the total neuronal population (McDonald, 1982), and interneurons (INs), which account for the remaining neuronal population (Wolff et al., 2014).

Within the first two postnatal weeks, LA INs and PNs are simultaneously contacted at the single cell level by long-range cortical and thalamic projections and undergo acute and chronic changes that are key events for the establishment of associative fear learning (LeDoux, 2000; Tsvetkov et al., 2002; Rumpel et al., 2005; Han et al., 2007, 2009; Humeau et al., 2007). Within the first postnatal week the LA cellular components undergo important developmental changes in circuit organization and physiological maturation of single cells (Berdel et al., 1997; Erlich et al., 2012; Ryan et al., 2016) so that, by the second postnatal week, the connections of the external capsule and the internal capsule within the LA neurons are fully established (Bouwmeester et al., 2002). According to this maturation profile, the mechanisms behind fear conditioning also evolve from the youth to the adult stage (Pattwell et al., 2012, 2016; Shoji et al., 2016). Although the data mentioned above suggest that the animal behavior and the intrinsic neuronal properties in LA are both developmentally regulated, so far the maturation of long-range projections to LA neurons has been investigated only in relation to excitatory pyramidal neurons (Gambino et al., 2010). The role of presynaptic proteins in these developmental processes is totally unknown. Within presynaptic proteins, the synapsins (Syns) are good candidates for playing a role in the maturation and connectivity of long-range projections to LA neurons. Syns are a family of abundant neuronal phosphoproteins encoded by three distinct genes (*Syn1, Syn2, and Syn3*) that play a central role in neuronal development, synaptogenesis and mature synaptic transmission and plasticity (Cesca et al., 2010; Fornasiero et al., 2010). The expression of Syns increases during postnatal developmental, overlapping with the peak of synaptogenesis (Bogen et al., 2009). Syns participate in the development of young neurons, by interacting with cytoskeletal and vesicular components, and influence axon elongation and synapse formation. Moreover, SynI plays a role in adjusting the number of synaptic connections in response to extracellular signals in a phosphorylation-dependent fashion (Perlini et al., 2011). Although Syns do not appear to be essential for gross brain embryogenesis, the proteins are essential for proper neuronal development and function, as demonstrated by the phenotype of Syn knockout (KO) mice and by the human pathologies associated with SYN1 mutations (Garcia et al., 2004; Fassio et al., 2011; Lignani et al., 2013; Nguyen et al., 2015). Indeed, deletion of Syns impacts on multiple forms of synaptic plasticity in both vertebrate and invertebrate preparations (for a review, see Cesca et al., 2010) and, consistently, behavioral experiments have shown that fear conditioning, emotional memory and cognition fear responses are influenced by the expression of Syns (Corradi et al., 2008; Porton et al., 2010).

Here, we first tested the hypothesis that a fundamental part of synaptic maturation occurs at the level of cortical and thalamic long-range projections to PNs and INs in LA with an input-specific pattern. Indeed, by performing simultaneous recordings of LA-INs and LA-PNs in WT mice, we demonstrate that adult projections show a strengthening of the synapses to PNs, but not to INs. Then, we studied the possibility that the development and maturation of connectivity in the LA depends on the postnatal expression of presynaptic proteins, such as the most abundant Syn isoform, SynI, by studying the functional impact of its deletion on the functional maturation of LA neuronal circuits.

The data demonstrate that SynI plays a fundamental role in age-related synaptic maturation. SynI absence only in putative excitatory synapses projecting to excitatory neurons causes a deficit of synaptic strengthening of specific intra-LA connection on adult animals which is a reflection of an incomplete synaptic evolution during development. Together, these results underline the fundamental importance of SynI in the maturation of synaptic connectivity in LA that may underlie neuronal circuit functionality and animal emotional behavior.

## Materials and Methods

### Animals

SynI KO mice were generated by homologous recombination (Chin et al., 1995) and backcrossed on C57BL/6J mice for more than 10 generations. SynI KO/GAD67 GFP-positive mice were obtained by crossing constitutive SynI KO mice with GAD67-eGFP mice (C57BL/6J background) kindly provided by Dr. A. Luthi (FMI, Basel, Switzerland) (Tamamaki et al., 2003). Hemizygous KO male mice and WT littermates aged from 2 weeks to 7 months were used. All experiments were performed in accordance with the guidelines established by the European Communities Council (Directive 2010/63/EU of September 22, 2010) and were approved by the Italian Ministry of Health and by Animal Experimental Committee of Bordeaux Universities (CE50; A5012009).

### Solutions

The solution compositions were the followings: NMDG-HEPES recovery solution: NMDG 93 mM; HCl 93 mM; KCl 2.5 mM; NaH_2_PO_4_ 1.2 mM; NaHCO_3_ 30 mM; HEPES 20 mM; Glucose 25 mM; Sodium ascorbate 5 mM; Thiourea 2 mM; Sodium pyruvate 3 mM; MgS0_4_.7H_2_O 10 mM; CaCl_2_ 0.5 mM; N-Acteyl-L-cysteine, 12 mM. ACSF-HEPES solution: NaCl 92 mM; KCl 2.5 mM; NaH_2_P0_4_ 1.2 mM; NaHCO_3_, 30 mM; HEPES 20 mM; Glucose 25 mM; Sodium ascorbate 5 mM; Thiourea 2 mM; Sodium Pyruvate 3 mM; MgSO_4_.7H_2_O 2 mM; CaCl_2_ 2 mM; N-acetyl-L-cysteine 12 mM. ACSF: NaCl 124 mM; KCl 2.5 mM; NaH_2_PO_4_ 1.2 mM; NaHCO_3_ 24 mM; HEPES 5 mM; Glucose 12.5 mM; MgS0_4_.7H_2_O 2 mM; CaCl_2_ 2 mM.

### Acute Brain Slices Preparation

The dissection method was adapted as previously described (Houbaert et al., 2013). Briefly, two beakers were filled with oxygenated *N*-methyl-D-glucamine (NMDG)-HEPES recovery solution, one on ice (4°C) and the other at 33°C. Mice were anesthetized with a mixture of ketamine/xylazine and, after opening of the chest cavity, the heart was exposed and the cardiac muscle perfused with 10 ml of cold NMDG-HEPES recovery solution. Animals were decapitated, the brain removed and placed in 4°C NMDG-HEPES recovery solution for an additional minute. Then, 300 μm slices were cut and immediately incubated in 33°C bubbled HEPES recovery solution. Slices were transferred at room temperature for 60 min and maintained in HEPES holding solution throughout the day. Standard recording ACSF was used for recording (Humeau et al., 2003; Houbaert et al., 2013).

### Patch-Clamp Recordings

Dual whole-cell patch-clamp recordings were performed from amygdala neurons that were visually identified by infrared videomicroscopy using an upright microscope equipped with a 60× objective (Olympus, BX51). LA-INs were further identified using GFP expression revealed by sample illumination with blue light (470 nm, Olympus, TH-200). Freshly prepared acute slices were positioned in a recording chamber maintained at 33°C and continuously perfused with bubbled ACSF solution. Recordings were performed with microelectrodes made of borosilicate glass (3–5 MΩ, Harvard apparatus 30-0062 capillaries GC150T-10) connected with a multi-clamp amplifier device (Axon CNS Multiclamp 700B). Each electrode was precisely positioned in X/Y/Z axis using micromanipulators (Luigs and Neumann, SM7). Each analogic signal was corrected from 50 to 60 Hz noise using active filters (Hum Bug, Quest Scientific, Canada) before being digitized (Digidata 1440, axon instruments). Acquisition of cellular currents was performed using PClamp-10 (Molecular Devices). Signals were acquired at 10 kHz and filtered at 2 kHz.

### Electrophysiological Configurations and Protocols

**Figure 1:**
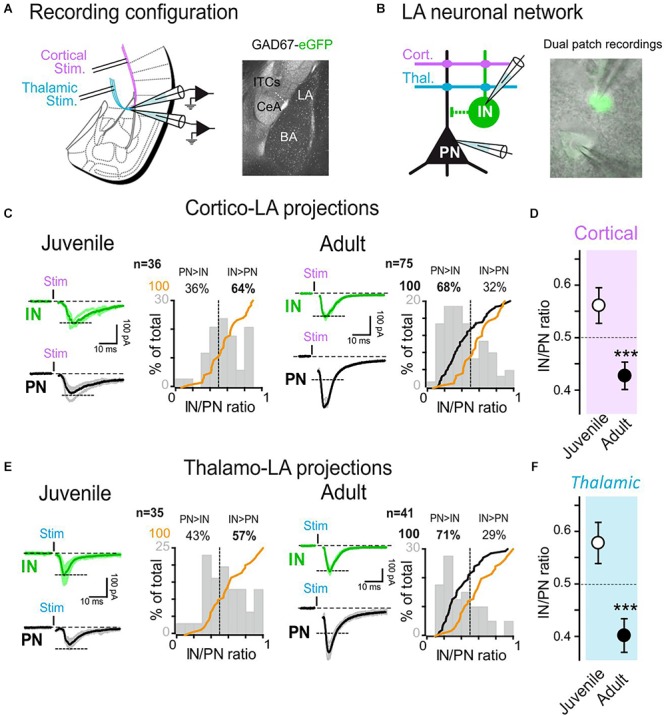
Target specific synaptic maturation occurs at long-range projections to the lateral amygdala during animal aging. **(A)** Left: Dual whole-cell recordings were obtained from LA cells in coronal acute slices. Two stimulation electrodes were positioned in the external and internal capsules to stimulate cortical and thalamic inputs to the LA. Right: anatomical location of LA within the amygdala nuclei. **(B)** GFP-expressing LA-INs were identified and recorded together with a neighboring LA-PN. **(C)** Cortical-LA EPSCs were evoked in juvenile (2–3 weeks, *n* = 36 dual recordings) and adult (3–6 months, *n* = 75 dual recordings) WT mice and the IN/PN ratio calculated. Typical traces are shown on the left panels and IN/PN ratios are shown on the right. The orange lines denote the cumulative IN/PN values for the juvenile dataset, and the black line the results obtained in adult mice. **(D)** Plot of Juvenile vs. Adult IN/PN ratio for cortical afferents. ^∗∗∗^*p* < 0.001, Mann–Whitney U-test. **(E)**: Same presentation as in **(C)** for thalamic-LA stimulations. **(F)** Plot of Juvenile vs. Adult IN/PN ratio for thalamic afferents. ^∗∗∗^*p* < 0.001, Mann–Whitney U-test.

All experiments consisted in simultaneous recordings of one LA principal neuron (LA-PN) and one LA interneuron (GFP-positive LA-IN) separated by less than 50 μm. The PN and IN were recorded with different intracellular solutions, based on the physiological objective: PN cells were recorded only in voltage-clamp mode at both -70 and 0 mV to record evoked excitatory and inhibitory transmission, respectively. The recording pipette was filled with a low chloride solution containing (in mM): 140 Cs-methylsulfonate, 5 QX314 Cl, 10 HEPES, 10 phosphocreatine, 4 Mg-ATP, and 0.3 Na-GTP (pH adjusted to 7.25 with CsOH, 300 mOsm). INs were only recorded at -70 mV, using an electrode filled with an equimolar K-gluconate solution. To stimulate incoming fibers, two additional external stimulation electrodes (Phymep bipolar electrodes connected to an externally triggered ISO-Flex isolated stimulator) were positioned using mechanical micromanipulators either in the external or in the internal capsule (see Figure 1A) to stimulate cortical and thalamic fibers, respectively.

### Stimulation Protocols

While performing dual whole-cell patch-clamp recordings from LA-INs and LA-PNs, we recorded a number of neuronal and synaptic parameters, using dedicated stimulation protocols, as follows. *Cell capacitance*: an approximation of the cell size was obtained in VC by measuring the τ of the capacitive current recorded during the seal test. Seal tests were triggered by a 20 m sec-10 mV hyperpolarizing current applied at -70 mV. *Evoked excitatory transmission*: 1 m sec-long current pulses were applied through external stimulation electrodes to stimulate axonal projections from either cortical or thalamic areas. The amplitude of the stimulation pulse was set to elicit a minimal response as close as possible to a monosynaptic response of 100 pA in the LA-IN. Basal transmission was evoked at 0.1 Hz. *Spiking discharge:* In current-clamp mode, LA-INs were maintained at -70 mV, and current steps of 400 msec and various amplitudes (-50, 50, 150, 250, and 350 pA) were applied. The number of generated spikes was then counted. *IN-PN connectivity*: In current-clamp mode, supra-threshold depolarization steps were applied to the recorded INs, while the PN was maintained at 0 mV in the voltage-clamp mode. Average eIPSC amplitudes were obtained from single pulses or stimulation trains (5 stim, 20 Hz). *Minimal stimulations*: 100 consecutive close-to-threshold (in INs) stimulations were applied at 0.5 Hz through the stimulation electrodes. Stimulation intensity was chosen to equilibrate failure/success recorded in the IN. The mean amplitude and the frequency of failure/success in the simultaneously recorded PN were then calculated. *Input/Output curves:* At both cortical and thalamic afferents, we applied stimulation steps of increasing amplitude to compare synaptic strength under unbiased conditions. To achieve that, a minimum of 3 recordings were obtained and averaged for each stimulation intensity value. Stimulation pulses were of 1 m sec.

### Histology and Immunofluorescence

Animals were deeply anesthetized with an intraperitoneal injection of urethane and transcardially perfused with ice-cold 0.1 M phosphate buffer (PB; pH 7.4) until the liver became clear, followed by 4% paraformaldehyde in 0.1 PB. After perfusion, brains were briefly dissected and post-fixed in the same fixative solution overnight at 4 °C. After several washes in 0.1 M PB, brains were then cryoprotected by immersion in 10, 20, and 30% sucrose solutions and subsequently cut in 30 μm sections with a vibratome and stored at -20°C in a solution containing 30% ethylene glycol and 20% glycerol in 0.1 M PB. Sections containing lateral amygdala were then washed in phosphate-buffered saline (PBS, pH 7.4) and processed for free-floating immunofluorescence. After blocking step in PBS containing 0.05% Triton X-100 and 10% normal goat serum (NGS), sections were incubated overnight at room temperature with the following primary antibodies: rabbit anti-VGAT (1:250, Synaptic System), guinea pig anti-VGLUT1 (1:250, Synaptic System), mouse anti-Syn1 (mAb10.22). Antibodies were diluted in PBS with 3% of NGS and 0.05% Triton X-100. Double immunofluorescence (VGAT-SYN1 and VGLUT-SYN1) was performed with the simultaneous addition of the primary antibodies. Sections were then washed in PBS (4 × 10 min) and incubated for 1 h at 25°C with anti-mouse Alexa Fluor 568, and anti-rabbit Alexa Fluor 647 (Invitrogen, United States) or anti-guinea pig Alexa Fluor 647 (Invitrogen, United States). After several PBS rinses, sections were mounted on glass slide and observed with a Leica SP8 confocal microscope (Leica Microsystem, Germany). Z-series stacks of seven consecutive confocal sections (1024 × 1024 pixels) for a total depth of 2 μm of tissue were acquired at 40× using the multi-track mode to avoid fluorescence crosstalk (pinhole: 1.0 airy unit) and background labeling was subtracted. For the analysis of putative inhibitory synapses, VGAT puncta were counted and then co-localized with Syn1 puncta by means of Colocalization plug-in on Fiji software. Colocalized particles were then counted again. For the analysis of putative excitatory synapses, VGLUT puncta that colocalize or not colocalize with GFP signal were counted and then colocalized with SYN1 puncta.

### Statistics

When comparing the effect of one factor in a group, the Fisher exact test was used. The Fisher’s exact test was performed on raw data and the data were represented as % for simplicity. When data did not follow a normal distribution, we applied the Mann-Whitney rank-based U test. For Figure 4, 5, a 2-way ANOVA followed by the Bonferroni *post-hoc* test was used to test for the influence of both age and genotype. For all tests, statistical difference was set at *p* < 0.05 and analyses were performed using Sigmaplot 12 (Systat Software, Inc.,). Non significant *p* values are shown in Supplementary Table 1.

## Results

### Target-Specific Synaptic Maturation Occurs at Long-Range Projections to the Lateral Amygdala During Postnatal Development

To analyze the development of the functional organization of amygdala networks, we simultaneously recorded excitatory events from LA PNs and GFP-labeled INs as a function of age by stimulating either cortical or thalamic inputs.

In order to characterize how and to what extent long-range projections contacted LA over age, we compared the amount of excitatory current received simultaneously by PNs and INs in 2–3 weeks (juvenile) and 3–6 months old (adult) WT/GAD67 mice while stimulating the external capsule (cortical inputs) or the internal capsule (thalamic inputs) projections (Humeau et al., 2005; Figure 1A,B). Initially, using seal tests-based capacitance calculations, we confirmed that fluorescence expression was indeed segregating two neuronal populations of different size. Furthermore, pair connectivity experiments (see, e.g., Figure 3, 6) showed that presynaptic stimulation of the GFP-expressing cells was solely eliciting outward GABAergic currents in the PN when maintained at a membrane potential of 0 mV. Finally, GFP-expressing cells exhibited spiking patterns typical of GABAergic cells (Houbaert et al., 2013), showing that we were indeed performing simultaneously recordings from PNs and INs.

To compare results obtained from different animals, stimulation intensities delivered at cortical-LA and thalamic-LA afferents were set to elicit inward AMPA_R_ current amplitudes of about 100 pA in the INs (Figure 1C,E). Then, the currents elicited simultaneously in the recorded PN were quantified, allowing the comparison of the IN/PN ratio (current amplitude recorded from IN/current amplitude simultaneously recorded from PN). Interestingly, the IN/PN ratio was evolving with animal age, suggesting a major postnatal maturation. At both cortical-LA and thalamic-LA afferents, we observed that the IN/PN ratio was shifted toward LA-INs in juvenile mice (juvenile IN/PN balance: cortical, 0.56 ± 0.03; thalamic, 0.58 ± 0.04), whereas it shifted toward PN excitation in adult mice (adult IN/PN balance: cortical, 0.43 ± 0.03; thalamic, 0.40 ± 0.03; *p* < 0.001 at both afferents, Mann–Whitney U-test) (Figure 1D,F). These results suggest that synaptic maturation processes modify long-range projections to LA in a target-specific manner.

### Minimal Stimulation Paradigms Demonstrate a Strengthening of Long-Range Projections to Lateral Amygdala PNs During Postnatal Development

**Figure 2:**
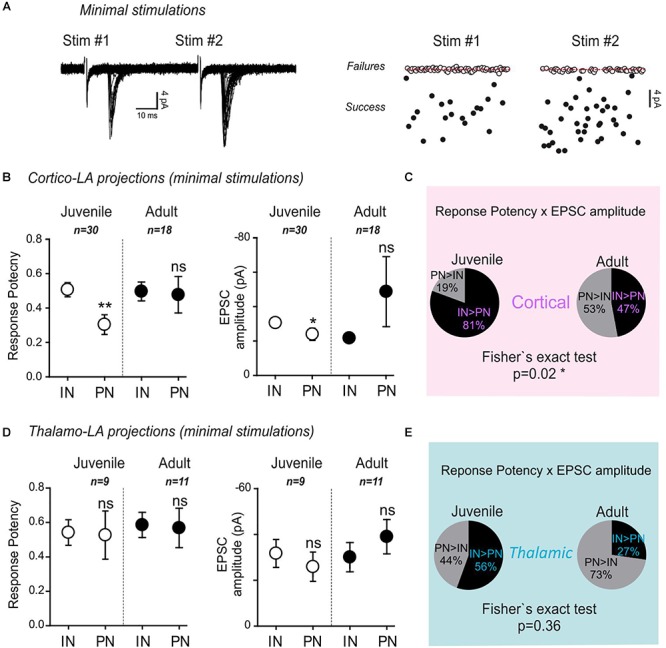
Minimal stimulation paradigms demonstrate a strengthening of long-range projections specifically to LA-PNs in adult mice. **(A)** Example of a minimal stimulation recording. Typical traces (left) and extracted EPSC amplitudes for 80 consecutive paired stimulations (#1 and #2) from the same recording (right). Note that the frequency of success increases at the second stimulation (stim#2) without changing the success amplitude. **(B)** Mean response potency (±SEM) and EPSC amplitude (±SEM) were extracted from INs and PNs in dual recordings of juvenile and adult preparations following stimulation of cortical axons. IN vs. PN: ^∗^*p* < 0.05; ^∗∗^*p* < 0.01; ns- *p* > 0.05; Mann–Whitney U-test. Green and black open dots: juvenile IN and PN, respectively; green and black close dots: adult IN and PN, respectively. An “excitatory drive” index was then calculated for each recording by multiplying the probability of success and the EPSC amplitude observed in simultaneously recorded IN and PN. **(C)** Pie charts representing the excitatory drive for cortical projections to PNs and INs. The percentage of the PN-excitatory drive over the IN-excitatory drive, and the IN-excitatory drive over the PN-excitatory drive calculated in paired recordings is shown. 50% represent the perfect balance between the excitatory projections to PNs and INs. **(D)** Same representation as in **(B)** for thalamic axon stimulations. INs vs. PNs ns- *p* > 0.05; Mann–Whitney U-test **(E)** same representation as in **(C)** for stimulation of thalamic axons.

The observed age-dependent change in the IN/PN ratio could originate from a decrease of excitation on INs, an increase of excitation on PNs, or a combination of the two. To separate between these scenarios, we performed minimal stimulation recordings to assess IN/PN ratio at the level of putative single axons (Tsvetkov et al., 2002; Jayachandran et al., 2014). The stimulation intensity was set to elicit postsynaptic currents in the recorded IN with a 50% success probability. Under these conditions, the postsynaptic responses were consistently stable in amplitude and, when a second stimulation was applied with a 50 msec delay, the probability of success mainly increased, indicating that glutamate release was likely mediated by a single axon (Figure 2A). We then measured probability and amplitude of excitatory responses occurring in INs and PNs at both cortical (Figure 2B,C) and thalamic afferents (Figure 2D,E).

In juvenile mice, both the response potency (INs: 0.51 ± 0.04; PNs: 0.3 ± 0.06; *p* = 0.002, Mann–Whitney U-test) and the minimal EPSC amplitude (INs: -30.3 ± 2.6 pA; PNs: -23.7 ± 3.6; *p* = 0.04, Mann–Whitney U-test) upon stimulation of cortical-LA projections were globally lower in PNs than in INs (Figure 2B).

Noteworthy, both response potency and EPSC amplitude were not significantly different between PNs and INs in adult mice. When these two parameters were combined to yield the excitatory drive impinging on PNs and INs, respectively (excitatory drive = EPSC amplitude x success probability), a significant shift toward a stronger excitatory drive on PNs was observed in adult mice (Cortical Excitatory drive: Juvenile, PN > IN 19%, IN > PN 81% *n* = 30; Adult, PN > IN 53%, IN > PN 47% *n* = 18; *p* = 0.02, Fisher exact test) (Figure 2C). On the contrary, no significant effects were found at thalamic-LA projections (Figure 2D,E) (Thalamic Excitatory drive: Juvenile, PN > IN 44%, IN > PN 56% *n* = 9; Adult, PN > IN 73%, IN > PN 27% *n* = 11; *p* = 0.36, Fisher exact test).

These data collectively show that target-specific, age-dependent modifications affect cortical long-range projections to the LA characterized by a strengthening process leading to an overall increase in excitatory drive specifically on PNs.

### The IN-PN Connectivity Remains Stable During Postnatal Development

**Figure 3:**
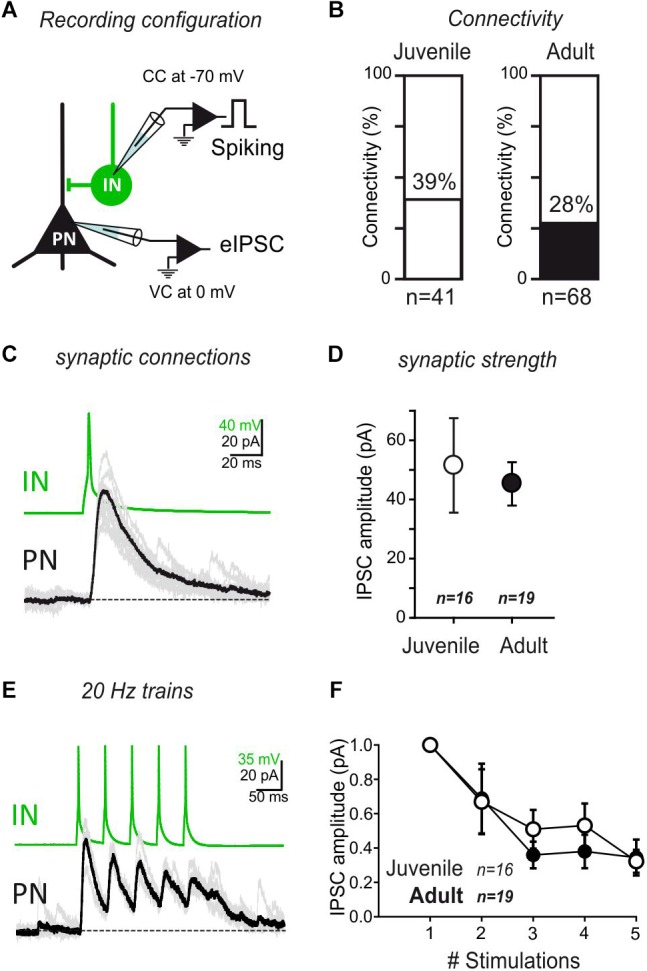
IN/PN connectivity is stable during development. **(A)** Connectivity between INs and PNs was tested by evoking action potentials in the INs, while recording the PNs at a membrane potential allowing visualizing GABA_A_ currents as outward currents. **B** The rate of connectivity was similar between juvenile and adult preparations. Juvenile: 39% *n* = 41; Adult: 28% *n* = 68. *p* = 0.13, Fisher exact test. **(C)** Single spike recording of a monosynaptic connection. **(D)** Average IPSC amplitude (±SEM) of IN/PN connections in juvenile and adult preparations for single spike stimulation. ns- *p* > 0.05; Mann–Whitney U-test. **(E)** Train stimulations of IN/PN connections (5×; 20 Hz intra burst). **(F)** Average IPSC amplitude (±SEM) of IN/PN connections in juvenile and adult preparations for 20 Hz train stimulation. ns- *p* > 0.05; 2-way ANOVA across age.

Next, we used simultaneous recordings of both INs and PNs to test synaptic connectivity in juvenile and adult mice. INs were depolarised to generate action potentials, while the resulting GABAergic current was recorded in a neighboring PN (Figure 3A). Interestingly, the level of connectivity and the mean amplitude of the synaptic responses were comparable between juvenile and adult mice (Figure 3B–D) (Connectivity juvenile: 39% *n* = 41; Adult: 28% *n* = 68; *p* = 0.13, Fisher exact test. EPSC amplitude Juvenile: 51.76 ± 16.13; Adult: 45.51 ± 7.29; *p* > 0.05, Mann–Whitney U-test). Then, using five repeated stimulations at 20 Hz, both juvenile and adult synapses exhibited similar depression rates (Figure 3E,F; *p* = 0.67, 2-way ANOVA juvenile vs. adult). These results show that, although the excitatory drive provided by long-range projections is strongly age-dependent, the IN to PN intra-connectivity in the LA is globally stable over development.

### Synapsin I Deletion Impairs Postnatal Synaptic Maturation in the Lateral Amygdala

It has been previously shown that thalamic and cortical long-range projections to the amygdala are of critical importance for fear acquisition, and that these synapses change with age (Quirk et al., 1997; Tsvetkov et al., 2002). The data shown so far demonstrate that from young to adult brain specific reinforcements occur onto PN synapses in the lateral amygdala. One of the synaptic proteins that was shown to be important in fear acquisition is SynI, which expression levels increase over aging and following this form of aversive Pavlovian conditioning (Ota et al., 2010). Aged SynI KO mice develop some cued fear-conditioning deficits that are not present in juvenile animals (Corradi et al., 2008). Thus, we wanted to test whether SynI deletion could affect the observed synaptic maturation at long-range projections to the LA during postnatal development.

**Figure 4:**
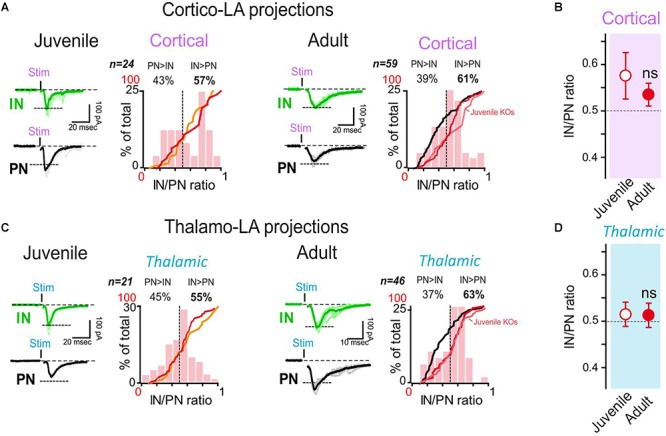
Synapsin I deletion does not change the LA organization in juvenile mice, but impairs synaptic maturation in adults. **(A)** Dual whole-cell recordings were obtained from LA cells in coronal acute slices from SynI KO mice. Cortical-LA EPSCs were evoked in juvenile (2–3 weeks, *n* = 24 dual recordings) and adult (3–6 months, *n* = 59 dual recordings) mice, and the IN/PN balance calculated. Typical traces (left) and IN/PN balances (right) are shown. Orange line: cumulative IN/PN values for the juvenile WT dataset; red line: results of the present data set; black line: results obtained in adult WT mice. WT groups are the same represented in Figure 1. **(B)** Averaged IN/PN ratio (±SEM) for cortical connections were calculated from juvenile and adult SynI KO mice. ns- *p* > 0.05; Mann–Whitney U-test. **(C)** Same presentation as in **(A)** for thalamic-LA stimulations. **(D)** Averaged IN/PN ratio (±SEM) for thalamic connections were calculated from juvenile and adult SynI KO mice. ns- *p* = 68; Mann–Whitney U-test.

To this aim, we crossed the SynI KO line with GAD67-eGFP reporter mice (WT/GAD67) to easily identify local INs (Houbaert et al., 2013). Then, we performed the above-described dual IN/PN recordings in juvenile and adult SynI KO/GAD67 mice and compared the results with WT/GAD67 littermates. Interestingly, juvenile SynI KO/GAD67 displayed a IN/PN ratio that was comparable to that found in WT/GAD67 mice (orange and red lines in Figure 4A,C) at both cortical and thalamic afferents (IN/PN ratio in juvenile SynI KO/GAD67: cortical, 0.58 ± 0.05, *p* > 0.05 Mann Whitney U test; thalamic, 0.53 ± 0.02; *p* > 0.05 Mann Whitney U test) (Figure 4B,D). These results confirmed that SynI does not play a major role in the assembly of synaptic contacts between these brain regions at the juvenile stage.

We then performed the same experiments in adult SynI KO/GAD67 mice (Figure 4A–D). While a clear shift in the IN/PN balance was observed in adult WT/GAD67 mice (IN/PN balance juvenile vs. adult WT/GAD67: *p* < 0.05 at both afferent, 2-way ANOVA followed by the Bonferroni *post-hoc* test), the IN/PN balance remained virtually unaltered in adult SynI KO/GAD67mice (IN/PN balance juvenile vs. adult WT: *p* > 0.05 at both afferent, 2-way ANOVA followed by the Bonferroni *post-hoc* test) (Figure 4B). Indeed, adult SynI KO/GAD67 mice displayed a larger IN/PN ratio (61% and 63% for cortical and thalamic stimulations, respectively, Figure 4B,D) than WT/GAD67 mice (32% and 29% for cortical and thalamic stimulations, respectively, Figure 1E,F). Thus, adult SynI KO/GAD67 mice closely mimic the juvenile condition, indicating that the lack of SynI affects the physiological synaptic maturation of long-range projections on LA neurons.

### Synapsin I Deletion Affects Synaptic Maturation Only on PNs of Lateral Amygdala

**Figure 5:**
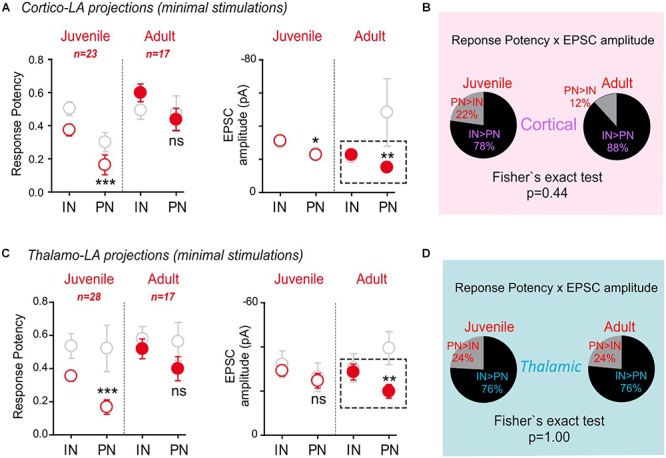
Synapsin I deletion blocks synaptic maturation at excitatory inputs onto LA-PNs in adult mice. Same as in Figure 2. **(A)** Mean response potency (±SEM) and EPSC amplitude (±SEM) were extracted from INs and PNs in dual recordings in juvenile and adult SynI KO preparations following stimulation of cortical axons. IN vs. PN: ^∗^*p* < 0.05; ^∗∗^*p* < 0.01; ^∗∗∗^*p* < 0.001; ns- *p* > 0.05, Mann–Whitney U-test. An “excitatory drive” index was then calculated for each recording by multiplying the probability of success and the EPSC amplitude observed in simultaneously recorded INs and PNs. Red open dots: juvenile synIKO; red close dots: adult synIKO; light gray open dots: juvenile and adult WT (Figure 2). **(B)** Pie charts representing the excitatory drive in SynI KO for cortical projections to PNs and INs. The percentage of the PN-excitatory drive over the IN-excitatory drive, and the IN-excitatory drive over the PN-excitatory drive calculated in paired recordings is shown. 50% represent the perfect balance between the excitatory projections to PN and IN. **(C)** Same as in **(A)** for stimulation of thalamic axons. IN vs. PN: ^∗∗^*p* < 0.01; ^∗∗∗^*p* < 0.001; ns- *p* > 0.05; Mann–Whitney U-test. **(D)** Same as in **(B)** for stimulation of thalamic axons.

Minimal stimulation recordings in WT/GAD67 mice revealed that the age-dependent shift in the IN/PN ratio in LA mostly relies on an increase of the excitatory response potency to PNs (Figure 2). To examine whether the lack of this phenomenon is at the basis of the maturation impairment observed in the absence of SynI, we repeated minimal stimulation experiments in juvenile and adult SynI KO/GAD67 mice (Figure 5). In accordance with the similar IN/PN ratio, no major differences were observed in juvenile SynI KO/GAD67, as compared to their WT/GAD67 littermates (Figure 5A,C, 2B,D). Nevertheless, a decrease in the response potency at thalamic-PN synapses was observed as compared to IN synapses (INs: 0.37 ± 0.03; PNs: 0.17 ± 0.05, *p* = 0.002, Mann–Whitney U-test), possibly linked to small differences in the success rate (set to 50%) after minimal thalamic stimulation in juvenile SynI KO/GAD67 and WT/GAD67 mice.

Interestingly, opposite to what was observed in WT/GAD67, adult SynI KO/GAD67 mice displayed decreased PN response amplitudes at cortical (IN: -22.9 ± 1.5 pA, PN: 15.2 ± 1.8 pA; *p* = 0.01, Mann–Whitney U-test) and thalamic (IN: -28.64 ± 3.6 pA, PN: 19.9 ± 3.1 pA; *p* = 0.01, Mann–Whitney U-test) projections as compared to IN responses. Remarkably, both projections lack an increase in the response potency on PNs in adult SynI KO/GAD67 mice (Cortical response potency: Juvenile, PN > IN 22%, IN > PN 78% *n* = 23; Adult, PN > IN 12%, IN > PN 88% *n* = 17; *p* = 0.44, Fisher exact test. Thalamic response potency: Juvenile, PN > IN 24%, IN > PN 76% *n* = 28; Adult, PN > IN 24%, IN > PN 76% *n* = 17; *p* = 1.00, Fisher exact test) (Figure 5B,D). Multiple mechanisms can explain why, PNs appears to be better targets for incoming long-range projections in WT mice: it can be proposed that LA-PNs develop early some determinants that make them preferential targets for incoming axons or, alternatively, that synapses onto these cells would have specific “reinforcing” mechanisms that would allow these synapses to be strengthened during animal life and experience. Another possibility is that synapses onto INs would have been generally reinforced, re-equilibrating the IN/PN balance because of IN changes. To separate between these possibilities, we first performed Input/Output (I/O) curves at both cortical and thalamic afferents (Supplementary Figure 1). Not surprisingly, as previously observed, the global relationship between stimulation intensity and response amplitude was different at cortical and thalamic synapses. Indeed, the evoked current was higher for thalamic stimulations (stim 1 mA: Thal. EPSC-PN: 690 ± 139 pA; Cort. EPSC-PN: 300 ± 69 pA, *n* = 26 and 29, respectively, *p* < 0.01 Mann-Whitney U-test) (Supplementary Figures 1B,C). Furthermore, a clear “saturation” of EPSC amplitude was observed at the highest thalamic stimulation, a phenomenon that is not present upon cortical stimulations, and classically, the first evoked EPSC was triggered at lower stimulation intensities for thalamic inputs than for cortical inputs. However, in SynI KO/GAD67, no difference was found between the thalamic and cortical evoked currents in IN and PN cells, all along the stimulation intensity range (Supplementary Figures 1B,C right panels). This confirmed that in absence of SynI, INs and PNs are equally contacted by incoming inputs. Interestingly enough, a direct comparison of EPSC amplitudes between genotypes suggested again that this difference originates in a lack of incoming excitation onto PNs: indeed, the mean current amplitude was systematically decreased in PN cells from KO mice (stim 1 mA: WT Thal. EPSC-PN: 690 ± 139 pA; SynIKO/GAD67 Thal EPSC-PN: 453 ± 93 pA; WT Cort. EPSC-PN: 300 ± 69 pA; SynIKO/GAD67 Cort. EPSC-PN: 115 ± 19 pA; *n* = 26, 25, 29, and 22, respectively, *p* < 0.01 between genotypes, Mann-Whitney U-test). No effect of the mutation on excitatory currents elicited in LA-INs by thalamic stimulations could be detected, and only a slight – but significant - decrease of EPSCs following cortical stimulations was detected WT Cort EPSC-IN: 201 ± 48 pA; SynIKO/GAD67 Cort. EPSC-IN: 98 ± 17 pA; *p* < 0.05, Mann-Whitney U-test (Supplementary Figure 1B). Nevertheless, PNs and INs I/O curve recordings also showed that SynI deficiency did not abolish some of the intrinsic thalamic/cortical differences: indeed, for all stimulation intensities, responses to thalamic stimulations were found higher than the cortical ones (stim 1 mA: SynIKO/GAD67 Thal. EPSC-IN: 340.5 ± 58 pA; SynIKO/GAD67 Cort. EPSC-IN: 98 ± 17 pA; *p* < 0.01, Mann-Whitney U-test).

These results demonstrate that synaptic maturation at cortical and thalamic projections depends on the expression of SynI. Furthermore, they also emphasize the importance of the strengthening of the excitatory input on PNs in mediating the age-dependent shift in the IN/PN ratio observed in WT/GAD67 mice.

### Synapsin I Deletion Does Not Affect the Stability of IN to PN Connectivity During Development in the Lateral Amygdala

**Figure 6:**
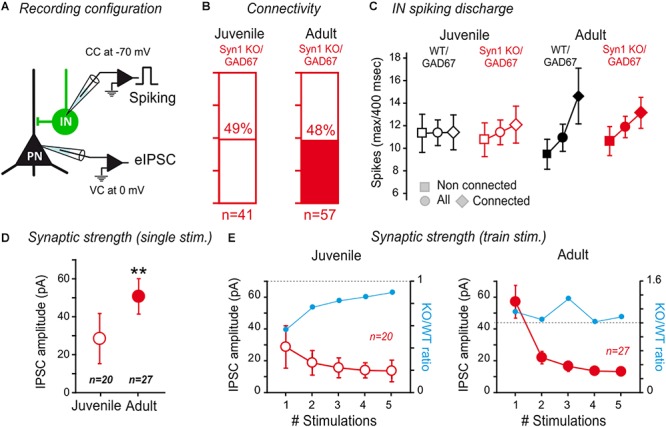
Synapsin I deletion does not affect IN to PN connectivity in the LA. **(A)** Connectivity between INs and PNs in SynI KO was tested by evoking action potentials in the INs, while recording the PNs at a membrane potential allowing to visualize GABA_A_ currents as outward currents. **(B)** The rate of connectivity was similar between juvenile and adult SynI KO preparations. Juvenile: 49% *n* = 41; Adult: 48% *n* = 57; *p* > 0.05, Fisher exact test. **(C)** Analysis of the maximum spike number (±SEM) (for a 400 m sec-long depolarization step) displayed in the INs of connected, non-connected or all tested pairs. See text for more details. **(D,E)** Properties of connected pairs. **(D)** Average IPSC amplitude (±SEM) of IN/PN connections in juvenile and adult SynI KO preparations for single spike stimulation. ^∗∗^*p* < 0.01; Mann–Whitney U-test. **(E)** Average IPSC amplitude (±SEM) of IN/PN connections in juvenile and adult SynI KO preparations for 20 Hz train stimulation. ns- *p* > 0.05; 2-way ANOVA juvenile vs. adult.

Because the inhibitory/excitatory balance of the inputs received by PNs may be of crucial importance for their involvement in the learning of aversive behaviors (Ehrlich et al., 2009), we tested whether the inhibition was also affected in adult SynI KO mice (Figure 6A). While the extent of IN-PN connectivity was not different between juvenile SynI KO/GAD67 and WT/GAD67 mice (juvenile SynI KO/GAD67 connectivity: 48% *n* = 41; juvenile WT/GAD67 connectivity: 39% *n* = 41; *p* = 0.50, Fisher exact test), a higher connectivity was observed in adult SynI KO/GAD67 with respect to adult WT/GAD67 mice (adult SynI KO/GAD67 connectivity: 48% *n* = 57; Adult WT/GAD67 connectivity: 28% *n* = 68; *p* = 0.02, Fisher exact test) (Figure 6B, 3). A difference in the level of connectivity could derive from the heterogeneity of the recorded INs. Indeed, fast spiking PV-expressing neurons are often highly connected to the adjacent PNs, thus opening the possibility that the results obtained in SynI KO slices were more influenced by this subpopulation of INs. However, when we compared the maximal number of spikes exhibited by connected vs. non-connected cells in juvenile animals, both groups of recordings were indistinguishable (Figure 6C). On the other hand, only adult WT/GAD67 displayed a higher spiking frequency in connected pairs (max spiking rate in WT adults: connected 14.6 ± 2.5; non-connected: 9.5 ± 1.3; *p* = 0.014, KO; *p* > 0.05, Mann–Whitney U-test). Furthermore, no differences in synaptic strength was observed in FS and no-FS inhibitory neurons stimulating cortical projections (Supplementary Figures 2A,B).

Then, we compared the amplitude of the synaptic response at IN-PN synapses using single pulses (Figure 6D) or short train stimulations at 20 Hz (Figure 6E), as previously described. We observed an increase in the amplitude of single connections between juvenile KO/GAD67 and adult SynI KO/GAD67 (KO/GAD67 IPSC amplitude: 52 ± 9 pA; Adult SynI KO/GAD67: 29 ± 13 pA, *p* = 0.004; Mann–Whitney U-test) (Figure 6D). Notably, the KO/WT ratio was significantly lower in response to the first stimulation (blue line in Figure 6E), returned back to equilibrium at the 5th stimulation, indicating that the slightly lower connectivity in juvenile depends on a decrease in release probability, rather than on a global decrease in synaptic connectivity. In adults, the ratio did not show any clear evolution within trains (Figure 6E). Thus, with the exception of a slight decrease in release probability in juvenile, and a slightly higher connectivity in adult mice, SynI deletion did not impact IN to PN connectivity in the LA.

### Synapsin I Is Preferentially Localized at Putative Excitatory Synaptic Sites Contacting Principal Neurons

**Figure 7:**
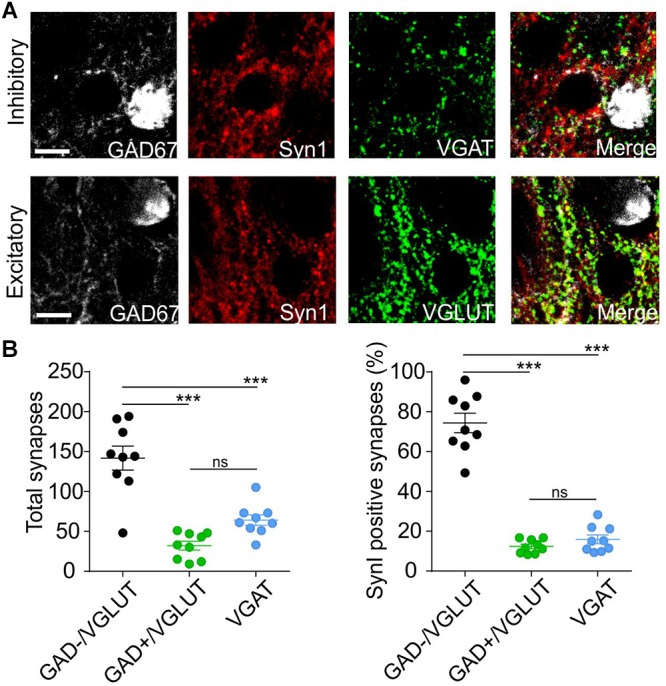
Synapsin I is mostly expressed in putative excitatory synaptic sites projecting to PNs. **(A)** Immunohistochemistry pictures for GAD67 (white), Synapsin I (red), vGAT (top, green), vGLUT (bottom, green), and merged channels in BLA. **(B)** Quantification of Synapsin I colocalization with vGLUT (putative excitatory synaptic sites on PNs), vGLUt and GAD67 (putative excitatory synaptic sites on INs) and vGAT (putative inhibitory synaptic sites). Left Total number of putative synaptic sites counted in 9 slices from 3 animals. ^∗∗∗^*p* < 0.001; ns- *p* > 0.05; one-way ANOVA followed by Bonferroni multiple comparison test. Right Percentage of putative synaptic sites co-localize with Synapsin I in the three groups counted in 9 slices from 3 animals. ^∗∗∗^*p* < 0.001; ns- *p* > 0.05; one-way ANOVA followed by Bonferroni multiple comparison test. Scale bar, 10 μm.

To examine whether endogenous SynI is present only in putative excitatory synapses contacting PN, coronal free-floating slices of LA neurons from adult GAD67 WT mice were stained with antibodies for SynI, vGAT (inhibitory synapses) and vGLUT (excitatory synapses) (Figure 7A and Supplementary Figure 3).

Immunofluorescence results show that SynI displays a high level of colocalization (up to 75%) with GAD-/vGlut+ puncta, representing putative excitatory contacts on PN. Instead, very low levels of colocalization in GAD+/vGlut+ puncta (excitatory contacts on IN) and vGAT puncta (inhibitory contacts) (Figure 7B). This result is in line with the electrophysiology data and it strengthened the thesis that postsynaptic identity determines pre-synaptic synapsin I expression.

## Discussion

The amygdala and its sub-nuclei are generally known for their prominent role in fear conditioning and as principal station for storing emotional memories. It is also well established how fear conditioning is the result of long-term changes depending upon macromolecular synthesis and calcium influx through NMDA channels (Blair et al., 2001). Yet, little is known for what concerns the synaptic mechanisms determining these long-term changes and how they are evolving through age. In this study, we explored the hypothesis whether afferent signals coming from the two principal sensory inputs to the lateral amygdala, the external and internal capsule, undergo synaptic strengthening during aging by dissecting short-term responses in the two principal LA populations. We performed an age-based comparison of the synaptic connectivity within the LA in WT mice by systematic pair recordings of GFP-expressing INs and neighboring PNs in acute slices. We also simultaneously examined IN and PN responsiveness to incoming external inputs and measured the amplitude response for single axon stimulations at the postsynaptic side (LeDoux, 2000; Maren, 2001; Humeau et al., 2007). Our results show a clear strengthening of the excitatory drive at both cortical/LA and thalamic/LA synapses onto PNs during physiological maturation of LA connectivity. We further identified as crucial mediator of this maturation the presynaptic protein SynI. Our data show that the developmental shift was virtually absent in SynI KO mice, identifying SynI as major responsible for the target-specific strengthening of long-range projections onto PNs that occurs under physiological conditions. Moreover, immunofluorescence results in GAD67-GFP mice suggested that SynI expression is restricted to putative excitatory synapses targeting PNs but not INs. This data show for the first time that SynI presynaptic expression is controlled by the postsynaptic identity, underlying its importance for plasticity phenomena. Notably, when we compared the paired-pulse ratio (PPR) of excitatory synapses on PN and IN, by stimulating the cortical afferents in both WT and SynI KO/GAD67 mice, we did not observe any difference (Supplementary Figures 2C,D). Although before, a decrease in eEPSC PPR was previously observed in SynIKO autapses, this effect was due to an increase of eEPSC amplitude and readily releasable pool (RRP) (Chiappalone et al., 2009). Here, we observed decreased eEPSCs on PN neurons in SynIKO mice compared to WT with no changes in PPR, but we did not look at RRP changes. We cannot exclude that, in the cortex or in other regions such as the hippocampus, SynI differently regulates the RRP, or that the *in vitro* experiments using autapses do not fully recapitulate what happens under physiological conditions.

A first novel finding is that the IN/PN ratio is age-dependent, with the progressive strengthening of excitation onto LA-PNs. The use of minimal stimulation paradigms allowed us to attribute this shift to the augmentation of single axon connections to LA-PNs. Although the data do not unambiguously demonstrate the quantal origin of this phenomenon, they strongly suggest that some sort of maturation and plasticity mechanisms occur solely at long-range inputs to amygdala PNs. Indeed, the postsynaptic sides of PNs and INs are functionally and morphologically different since E/E contacts are mostly located onto PN dendritic spines, while INs are almost devoid of postsynaptic spines. Dendritic spines are thought to restrict Ca^2+^ signaling and depolarization upon synaptic activation, but may also be important for the generation of feedback mechanisms through diffusive or *trans*-synaptic adhesion molecules. Further studies would be necessary to understand the contribution of postsynaptic or presynaptic effects in determining possible plastic changes at E/E and E/I synapses, but the experiments performed in SynI KO mice clearly indicate that presynaptic mechanisms are involved and are controlled by the postsynaptic side.

The electrophysiological evolution of LA circuits is consistent with the described morphological and cytoarchitectural changes of PNs and INs during maturation (Faber et al., 2001; Erlich et al., 2012; Ryan et al., 2012; Bosch and Ehrlich, 2015). The first two postnatal weeks represent a critical window in which dendritic development is particularly prominent (Ehrlich et al., 2013). The expansion of the dendritic arbor during the first postnatal period has been related to an increase of synaptic connections with projection neurons coming from the different capsules (McDonald, 1998). This would also potentiate the efficacy of multiple sensory stimuli on amygdala circuits, especially concerning the output behavior and fear learning and memory (Janak and Tye, 2015). Cortical projections to LA have been also characterized in several species, including rat, cat and monkey (McDonald, 1998). Differently from thalamic projections, which remain static with age, cortical projections undergo development and remodeling up to the first postnatal month (Bouwmeester et al., 2002). These processes are key events in amygdala physiology, as several studies have underlined the functional consequences of an impairment of cortical and subcortical projections on the input and processing of information into amygdala circuits, as well as on how emotions related to experience, memory and retrieval are stored (Phelps and LeDoux, 2005; Ryan et al., 2016).

Recently, progress has been made in the understanding of how local IN subpopulations control the PNs in the amygdala, with the emergence of complex disinhibitory circuits permitting to timely control somatic and dendritic inhibition during fear learning, expression and extinction (Wolff et al., 2014).

Functionally, the I/E ratio is set by the dynamic recruitment of local neuron populations and, more precisely, by the relative responsiveness of local INs and PNs to long-range inputs that are tightly organized in cortical structures to allow optimal information processing (Xue et al., 2014).

The crucial importance of Inhibitory/Excitatory (I/E) balance in information processing in the brain is illustrated by the numerous neurological conditions and cognitive disabilities associated with unbalances in discrete neuronal circuits (Gao and Penzes, 2015). However, in the vast majority of cases, the etiology of the changes remains unclear. In this scenario, SynI deficiency, which has been associated with I/E imbalances and identified as an epilepsy and autism predisposing gene in humans (Garcia et al., 2004; Fassio et al., 2011; Lignani et al., 2013; Nguyen et al., 2015; Peron et al., 2018) offers the possibility to explore amygdala-related pathogenic mechanisms under a SynI loss-of-function condition. Although SynI is known to differentially affect excitatory and inhibitory synapses at the presynaptic level (Cesca et al., 2010; Lignani et al., 2013; Medrihan et al., 2013), a distinction of the postsynaptic neuronal population has never been investigated.

*Ex vivo* work by Schafe and colleagues pointed out that the massive presynaptic rearrangements occurring in the LA following associative fear learning, are associated with a notable increase in SynI expression (Ota et al., 2010). These authors identified a retrograde signaling mechanism that may regulate fear memory consolidation at least partially through presynaptic mechanisms. As SynI is involved in several key steps of the SV cycle, at both pre- and post-docking levels, it is tempting to speculate that SynI-mediated mechanisms could potentially contribute to LTP consolidation and that, in the absence of SynI, the amygdala remains constrained in an “immature” juvenile state.

## Conclusion

In conclusion, the results provide a cellular basis for the SynI involvement in the maturation changes observed in the amygdala. We propose that SynI is a key regulator of synaptic strengthening in the LA, and that its absence impairs the physiological maturation of long-range excitatory projections to principal neurons in this brain region.

## Author Contributions

EL and ADF performed the experiments. YH and GL analyzed and interpreted the data. EL, GL, FB, and YH conceived and designed the work and drafted the manuscript.

## Conflict of Interest Statement

The authors declare that the research was conducted in the absence of any commercial or financial relationships that could be construed as a potential conflict of interest.
