# Supplementary material for: Synapsin I Controls Synaptic Maturation of Long-Range Projections in the Lateral Amygdala in a Targeted Selective Fashion
Source: Front Cell Neurosci. 2019 May 21;13:220. doi: 10.3389/fncel.2019.00220 (PMC6536628; doi:10.3389/fncel.2019.00220)
Supplement: Supplementary file 1 [file Data_Sheet_1.PDF]

Supplementary Figures and Tables

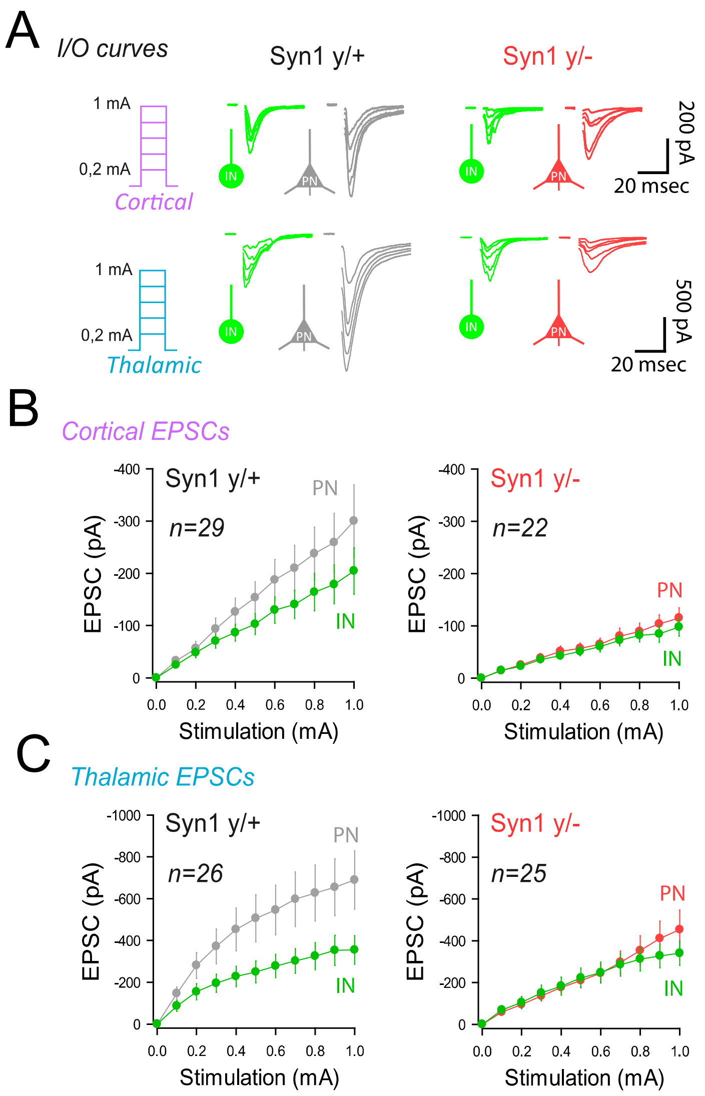

Supplementary Figure 1. Input/output protocol shows a predominant decrease of EPSC in pyramidal neurons in response to stimulation of both cortical and thalamic afferents. **a:** representative input/output protocol and relative traces for cortical and thalamic afferents in double-patched INs and PNs. **b,c:** PN and IN response at I/O curves during cortical (**b**) and thalamic (**c**) long-range projection stimulation. PN vs IN: Cortical Syn1 y/+ EPSCs from 0.7 to 0.9, *P<0.05, at 1mA, ***p<0.001 (n=27); Cortical Syn1 y/- EPSCs at 1mA, ns p<0.05 (n=19); Thalamic Syn1 y/+ EPSCs from 0.2 to 1mA, ***p<0.001 (n=23); Thalamic Syn1 y/- EPSCs at 1mA, ns p<0.05 (n=21). Two-Way ANOVA for repeated measurements followed by Bonferroni multi-comparison test.

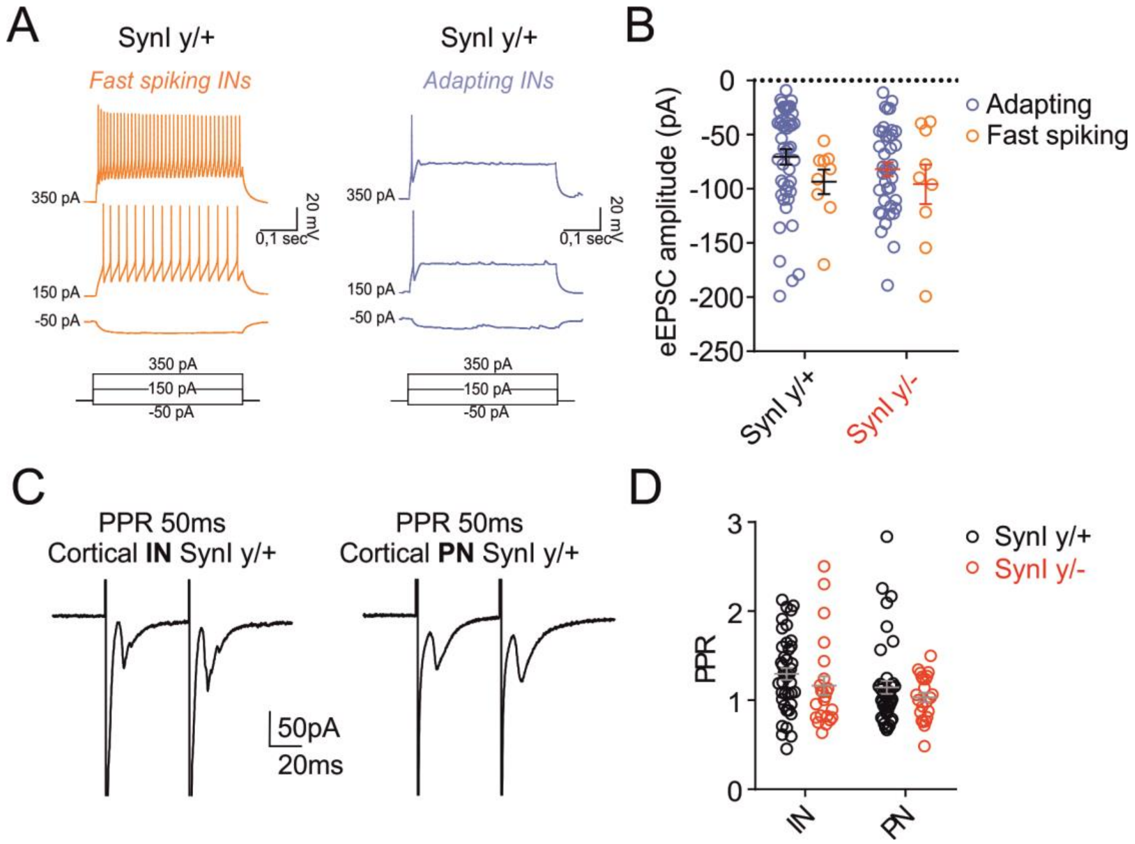

Supplementary Figure 2. The current received by FS and non-FS (adapting) INs upon stimulation of long-range afferents are not affected by lack of SynI in the lateral amygdala. **a:** representative traces and current injection of fast spiking and adapting INs. **b**: EPSC amplitude does not differ between FS and non-FS neurons in SynI y/+ and SynI y/-. **c:** representative traces of paired pulse stimulation in a SynI y/+ interneuron and pyramidal neuron. **d**: IN and PN paired-pulse ratio does not differ between SynI y/+ and SynI y/-. Ns, two-way ANOVA followed by Bonferroni multi-comparison test.

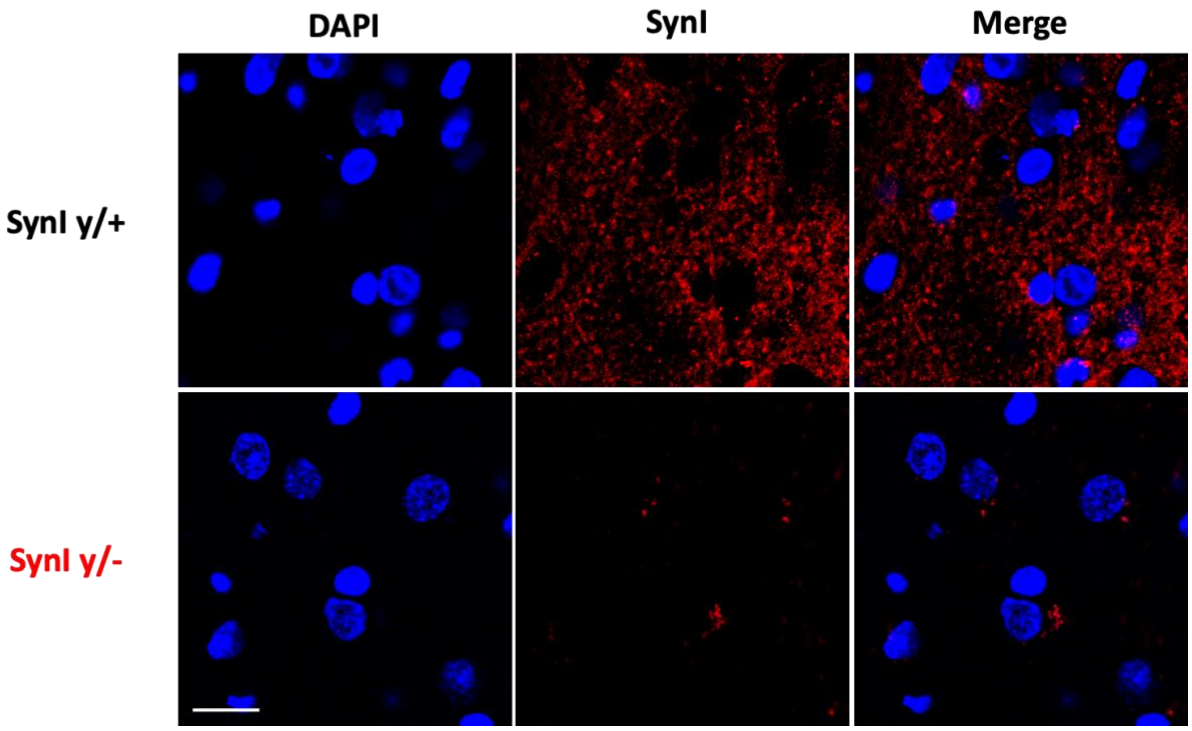

Supplementary Figure 3. Specificity of the SynI antibody used in SynI y/+ and SynI y/-. Immunohistochemical images for Dapi (Blue), SynI (Red) and merged channels in BLA showing the specificity of 10.22 anti-SynI antibody in WT (upper panel) and SynI KO (lower panel) animals. Scale bar, 20 µm.

Figure	Comparison	p
fig 2b (cort)	adult IN vs PN: success probability	0.91
	adult IN vs PN: amplitude	0.69
fig 2d (thal)	adult IN vs PN: success probability	0.34
	adult IN vs PN: amplitude	0.11
	juvenile IN vs PN: success probability	0.68
	juvenileIN vs PN: amplitude	0.74
fig 3d	IPSC amplitude synaptic strengh	0.41
fig 3f	IPSC amplitude stimulations	0.68
fig 4b (cortical)	IN/PN ratio in juvenile SynI KO	0.4
fig 4d (thalamic)	IN/PN ratio in juvenile SynI KO	0.68
fig 5a (cortical)	adult IN vs PN: success probability	0.062
fig 5c (thal)	adult IN vs PN: success probability	0.21
	juvenileIN vs PN: amplitude	0.16
fig 6b	connectivity	0.5
fig 7b	total synapses	0.096
fig 7 c	% positive synapses	>0.99
suppl fig 1b	Cortical SynIKO PN vs IN at 1mA	>0.99
suppl fig 1c	Thalamic SynIKO PN vs IN at 1mA	0.89

Supplementary Table 1. List of the non-significant p values referred to as p>0.05 in the various figures.
